# Winter feeding of elk in the Greater Yellowstone Ecosystem and its effects on disease dynamics

**DOI:** 10.1098/rstb.2017.0093

**Published:** 2018-03-12

**Authors:** Gavin G. Cotterill, Paul C. Cross, Eric K. Cole, Rebecca K. Fuda, Jared D. Rogerson, Brandon M. Scurlock, Johan T. du Toit

**Affiliations:** 1 Department of Wildland Resources, Utah State University, 5230 Old Main Hill, Logan, UT 84322, USA; 2 U.S. Geological Survey, Northern Rocky Mountain Science Center, 2327 University Way, Suite 2, Bozeman, MT 59715, USA; 3 U.S. Fish and Wildlife Service, National Elk Refuge, PO Box 510, Jackson, WY 83001, USA; 4 Wyoming Game and Fish Department, 432 Mill Street, Pinedale, WY 82941, USA

**Keywords:** *Brucella abortus*, brucellosis, chronic wasting disease, disease ecology, feedgrounds, wildlife–livestock conflict

## Abstract

Providing food to wildlife during periods when natural food is limited results in aggregations that may facilitate disease transmission. This is exemplified in western Wyoming where institutional feeding over the past century has aimed to mitigate wildlife–livestock conflict and minimize winter mortality of elk (*Cervus canadensis*). Here we review research across 23 winter feedgrounds where the most studied disease is brucellosis, caused by the bacterium *Brucella abortus*. Traditional veterinary practices (vaccination, test-and-slaughter) have thus far been unable to control this disease in elk, which can spill over to cattle. Current disease-reduction efforts are being guided by ecological research on elk movement and density, reproduction, stress, co-infections and scavengers. Given the right tools, feedgrounds could provide opportunities for adaptive management of brucellosis through regular animal testing and population-level manipulations. Our analyses of several such manipulations highlight the value of a research–management partnership guided by hypothesis testing, despite the constraints of the sociopolitical environment. However, brucellosis is now spreading in unfed elk herds, while other diseases (e.g. chronic wasting disease) are of increasing concern at feedgrounds. Therefore experimental closures of feedgrounds, reduced feeding and lower elk populations merit consideration.

This article is part of the theme issue ‘Anthropogenic resource subsidies and host–parasite dynamics in wildlife’.

## 1. Introduction

**Figure 1.**
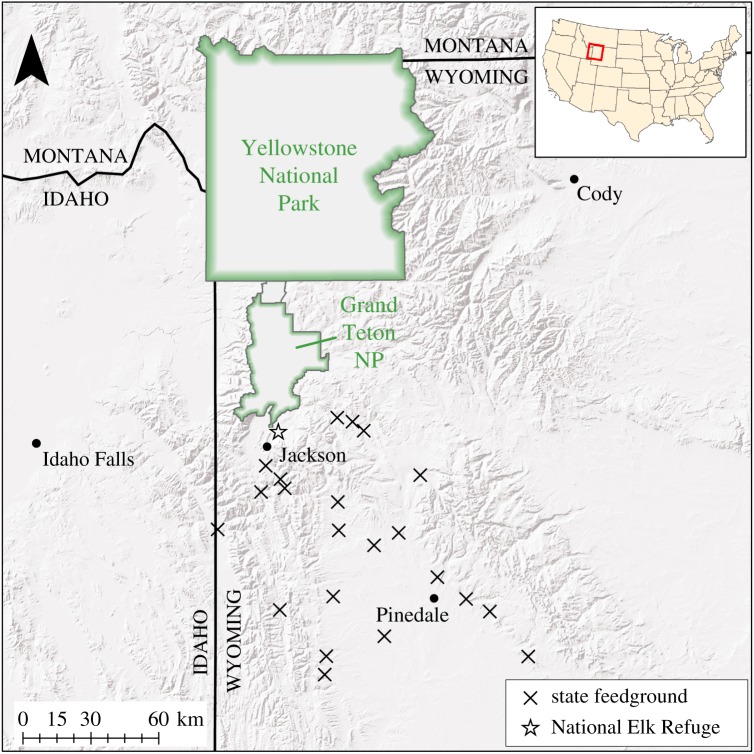
There are 23 supplemental feedgrounds for elk in Wyoming. The National Elk Refuge, north of Jackson, is operated by the US Fish and Wildlife Service, while the remainder are operated by the Wyoming Game and Fish Department. (Online version in colour.)

Central to many host–pathogen systems is the relationship by which infectious contacts increase with increasing host density. In wildlife, local aggregations often occur at sites of food provision, exemplified by winter feeding of elk (*Cervus canadensis*) at 23 locations across western Wyoming, USA. Unlike most anthropogenic food subsidies for wildlife, which exist incidentally (agricultural crops, garbage dumps) or intentionally in many small, widely dispersed loci (bird feeders), these feedgrounds are operated by government agencies and are used by an estimated 80% of the regional elk population [1]. Altogether, approximately 25 000 elk are fed on an annual basis [2]. Grass or alfalfa hay is generally provided *ad libitum* using horse-drawn sleighs except at the federally managed National Elk Refuge (NER), where pelleted alfalfa is dispensed from mechanized equipment due to the large number of elk that winter there. To our knowledge, this feedground complex (figure 1) represents the world's most concentrated institutional feeding programme for wildlife.

Institutional feeding began as early as 1907 and was formalized with the creation of the NER in 1912. The state of Wyoming assumed management of its first feedground in 1929 and the 22 they currently manage were mostly in place by the 1960s. They were initially established to support dwindling elk herds through the winter and provide a nutritional diversion from private haystacks, and remain popular with some sectors of the general public. They facilitate wildlife viewing and enhance sport-hunting opportunities (important sources of revenue), limit competition on winter ranges with other ungulates, mitigate some aspects of livestock conflict and locally offset winter starvation by elk. They are, however, implicated in disease concerns. Each feedground draws a herd of elk that congregates for weeks or months when individuals are perhaps most vulnerable to acquiring new infections. That feedgrounds facilitate disease transmission [3] has in itself created an additional reason for feeding elk—to separate them from cattle. Thus a cycle is perpetuated whereby feeding creates and mitigates the same problem: it enhances transmission among elk [3–6] while also limiting contact between elk and livestock in winter [7]. The way forward is murky and stakeholders should weigh the problems of feedgrounds maintaining disease against the opportunities of using them to adaptively manage disease. In this paper, we review the effects of winter feedgrounds on disease ecology with a focus on brucellosis in elk in western Wyoming. We also offer suggestions for future research and management.

## 2. Brucellosis in the Greater Yellowstone Ecosystem

For the past 50 years, much of the controversy surrounding the feedgrounds has focused on brucellosis. In the Greater Yellowstone Ecosystem (GYE), brucellosis is caused by the bacterium *Brucella abortus* and affects cattle, elk and bison (*Bison bison*). Globally it is an important zoonotic disease, but human cases in the USA are generally occupation-related and rare [8]. The greatest burden now imposed by brucellosis in the USA is economic. Brucellosis causes abortions and sterility in cattle so state and federal livestock regulatory agencies impose restrictions on sale and movement of infected cattle herds, which can reduce the profitability of affected and neighbouring herds [9].

### (a) Interspecific transmission among hosts

Bison and elk have contracted the disease from domestic cattle multiple times in the GYE since 1917 [10–12]. Although eradicated from cattle herds in the rest of the USA, brucellosis periodically spills back from elk to GYE cattle. Transmission occurs when susceptible animals have direct contact with aborted fetuses and other infective tissues and fluids [13,14]. Environmental persistence is relatively short-lived, as scavengers quickly remove infectious materials [5], although in cool, wet, shaded conditions the bacteria may remain viable for several months [15]. Thus, transmission requires either co-mingling or successive occupation of the same site within a limited time frame.

**Figure 2.**
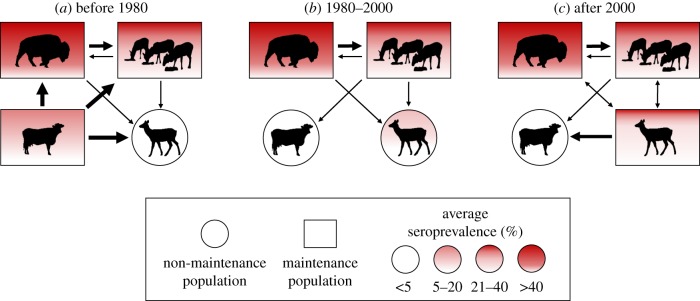
Hypothesized maintenance and reservoir hosts for *B. abortus* in the GYE during the three stages of the disease to date. Initially (*a*), cattle (bottom left) were a source population that infected bison (top left), fed elk (top right) and unfed elk (bottom right). After effective control measures were implemented in cattle (*b*) they were no longer a maintenance host but could be reinfected from fed elk. After 2000 (*c*), unfed elk became part of the reservoir community, able to maintain the infection in the absence of other host populations and are now a source of infection to cattle. Arrows depict established transmission paths. Arrow thickness denotes relative importance. (Online version in colour.)

The respective roles of elk and cattle as reservoirs for brucellosis have changed over time, whereas the role of bison appears to have remained constant (figure 2). Our understanding, though, has shifted. Prior to the 2000s, bison were considered the greatest risk to cattle because they exhibit higher disease prevalence (approx. 60%) than unfed elk (less than 5%), and while fed elk had higher seroprevalence (approx. 20%), they were separated from cattle by the feedgrounds [3]. Intensive management operations preclude bison and cattle co-mingling [16], and spillback events to cattle in the GYE have all been attributed to elk [10,17]. Nevertheless, bison remain an important maintenance reservoir. Interspecific transmission between bison and elk has been documented via whole-genome sequencing at the NER as well as in the free-ranging populations in Yellowstone National Park (YNP) [10], although there are currently insufficient data with which to estimate these rates. As a result, disease eradication is unlikely in one host without concurrent efforts across all hosts [18].

Similarly, prior to the 2000s there was broad consensus that free-ranging elk outside of the feedground complex were a non-maintenance population for brucellosis [14]. Although the spread of brucellosis in elk in most regions of the GYE traces back to the feedgrounds [10], more recently, it appears that higher levels of brucellosis seroprevalence in unfed elk herds unassociated with feedgrounds are self-sustaining, and in recent years there have been more cases of brucellosis in cattle away from, rather than in close proximity to, feedgrounds [6,7,16]. Because brucellosis prevalence is generally still higher among feedground than free-ranging elk, feedgrounds may reduce local transmission risk to cattle by facilitating elk–cattle separation.

### (b) Intraspecific transmission in elk

Captive studies have failed to demonstrate male-to-female sexual transmission in elk, cattle or bison [13,19,20], so that among- and within-species transmission have the same requirements with the possible exception of vertical transmission. Although calves born to infected elk exhibit a variety of outcomes, most are seronegative by 6 months of age [13]. Elk conceive in autumn and *Brucella*-induced abortions occur mainly during the third trimester due to mechanisms that are not perfectly understood (see [21]). About 50% of elk abort their first pregnancy following infection, after which the majority are thought to be recovered with immunity [13,22]. Winter feedgrounds operate between December and April, and *Brucella*-induced abortions peak between March and May [23]. Studies measuring contact and seroprevalence at different scales suggest that the probability of intraspecific transmission is correlated with elk density and aggregations along the feedlines [5,18,24].

**Figure 3.**
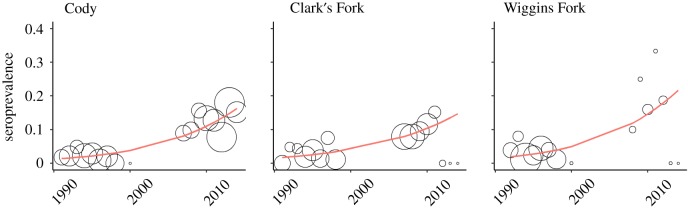
Seroprevalence trends from three unfed herd units in western Wyoming. Historically, unfed herds rarely exceeded 5% prevalence; however, seroprevalence of some unfed herds has crept upwards, now reaching the 10–40% seroprevalence observed on feedgrounds. Circle radius represents sample size; smoother lines were fit using generalized additive models with the mgcv package [25] in R [26]. (Online version in colour.)

Increased prevalence in unfed elk populations (figure 3) is similarly correlated with elk density and group size [6,27]. Elk group sizes in the GYE have a right-skewed distribution whereby most groups are small (e.g. fewer than 10), but most of the individuals in the population occur in groups of several hundred to several thousand [28]. These large groups probably play a disproportionate role in brucellosis maintenance and spread [6,18]. As unfed elk herds in the GYE have grown, so too have regional density and large winter aggregations associated with increasing brucellosis prevalence [27,29]. These large, unfed groups occur most frequently on private land or public land with late-season management closures where elk can escape hunting pressure, are larger on grasslands and even larger still on irrigated land [27,28]. Irrigated land may represent another form of anthropogenic food subsidy that is largely outside of management control and poorly studied in the context of disease ecology.

A potential hidden cost associated with feedgrounds is increased stress, which can increase disease susceptibility [30,31] and enhance intraspecific transmission. Forristal *et al*. [32] compared levels of faecal glucocorticoids (fGCs), a stress hormone, between fed and unfed elk during winter and found higher levels in fed populations. Feedgrounds differ in their localized elk density, predator densities and human activity, all of which could lead to elevated fGCs. Agonistic behaviour may also increase at feedgrounds as the result of elk sex- and age-class mixing that normally does not occur in winter.

Likewise, co-infections play an important role in the susceptibility, duration, transmission and expression of diseases [33–36]. Cytokines, which are cell-signalling proteins that mediate a host's anti-parasitic response, can be modulated by parasites themselves, and thus have been proposed as a useful way to gauge interactions like competition or synergism between co-infecting parasites [37]. Some evidence for synergism between *B. abortus* and the weakly pathogenic *Trypanosoma cervi*, with which Wyoming elk are chronically infected [38], has recently emerged [39]. Both appear to share a strategy in which they upregulate host production of the cytokine interleukin-10 (IL-10), which can impair immune response and facilitate chronic infections [40–43]. This effect may also hinder vaccine efficacy [39,43]. Any number of diseases carried by elk have the potential to interact in ways that are relevant to feeding. *Trypanosoma cervi* provides a useful illustration for this line of questioning that is relatively new to wildlife disease. Methods exist for quantifying elk cytokines using reverse transcription real-time polymerase chain reaction [44], and present a new approach to assess the effects of winter feeding on elk health.

### (c) Scavengers and predators

*Brucella*-induced abortions are infrequently detected on feedgrounds, in part because scavengers quickly consume or remove the fetuses [5,15,24]. Although transmission has occurred under experimental conditions, scavenger species are not thought to be important vectors for the spread or maintenance of brucellosis [45–48], and have the potential to mitigate transmission [18,49]. Coyotes (*Canis latrans*) are important fetal scavengers and feedgrounds have higher scavenging rates than unfed locations [5,15,24]. This has important implications for increasing prevalence of brucellosis in large, unfed aggregations of elk, as coyotes can be hunted year-round in most of the western USA, but benefit from relative protection at established feedgrounds. The effects of bears (*Ursus arctos* and *Ursus americanus*) on disease dynamics of the feeding grounds is unknown, but is probably minor because they are still hibernating for some of the transmission season.

Wolves (*Canis lupus*) have expanded their range since their reintroduction to Yellowstone National Park in 1995 and routinely kill elk at some feedgrounds. On several occasions wolves have chased elk off of feedgrounds, effectively halting feeding operations for most of the winter. Their impacts on feeding operations and winter elk survival at the NER have been minimal, but wolf presence is associated with an increase in the proportion of the Jackson herd that attend feedgrounds. Hunting by humans could have a similar effect, with most hunting occurring in the autumn and winter, and hunter avoidance by elk (movement to private land) being well documented in the western USA [50–52]. In areas where elk are sensitized to the risk of predation by both humans and wolves, elk may seek refuge at feedgrounds or on private land where hunting is restricted, leading to dense aggregations with increased brucellosis transmission risk.

The effect of wolves on winter aggregations of elk has yet to be fully explored. Wolf presence is associated with larger elk groups [28], but it is unknown whether wolves are simply following large numbers of elk, if the aggregation pattern represents a defensive behaviour in response to predators, or both. Finer spatial resolution is needed to assess the effect of wolves on brucellosis transmission in the context of winter feeding, because stagnant elk herds or elk returning to feedlines would be more likely to encounter fetuses compared with groups that spend more time on ‘fresh ground’ as a consequence of evading predators. The potential attraction of wolves to feedgrounds represents an additional concern to neighbouring ranchers where displacement of elk to private property may increase the spillback risk or result in incidental livestock depredation.

## 3. Adaptive management

Wildlife feeding programmes provide valuable opportunities for learning about disease dynamics in free-ranging populations. The GYE elk feedground system is currently operated—to the extent allowed by logistics, funding and politics—within an adaptive management framework to allow outcomes-based comparisons of alternative interventions [53]. Our experiences with this system underscore the need for *a priori* considerations of statistical design: treatments and controls applied with randomization, replication, stratification and calibration. Finally, alternative formulations of plausible models that account for a broad range of ecological and disease dynamic processes will speed up the learning process.

### (a) Vaccination

Feedgrounds offer enormous potential for vaccine delivery. The Wyoming Game and Fish Department (WGFD) began vaccinating feedground elk by airgun in 1985 [54] using a vaccine developed for use in cattle [55] and later used in elk [56]. By the time the programme ended in 2015, coverage exceeded 97% of elk calves, but there was no significant reduction in seroprevalence or abortion events [57]. Were an effective vaccine available, feedgrounds could facilitate an annual ‘doctor's visit’ to reduce contagion and risk. Unfortunately, the tools developed for use in cattle have yet to overcome either the immunological differences of elk, or some other unknown element such as co-infection [39]. Vaccine-development efforts are further constrained by the Select Agent status designated to *B. abortus* in the USA, which increases the regulations associated with handling live *Brucella* cultures. Should a new vaccine be developed, at least half of the female elk population would require vaccination [22], which may only be feasible where elk are fed. It is also vital that any new vaccine not impair surveillance efforts, i.e. that the vaccine strain be discernible from the pathogenic strain in serologic tests.

While empirical results ultimately contributed to the cessation of elk vaccination, the effects (or lack thereof) may have been more readily apparent with better implementation of experimental design. Initially there was only one control site and until recently there were no competing models to explain potential changes in seroprevalence (e.g. altered feeding seasons). In addition, treatments could have been phased in or out over different years across sites in order to control for annual variation and allow for cleaner pre–post comparisons.

### (b) Test-and-slaughter

**Figure 4.**
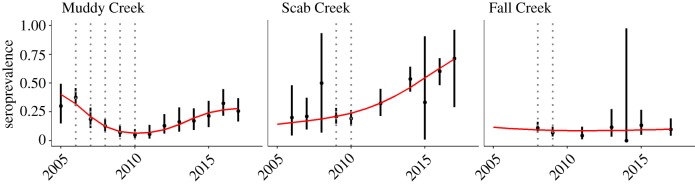
Test-and-slaughter of seropositive female elk between 2006 and 2010 reduced seroprevalence for brucellosis at the Muddy Creek feedground from 37% to 5%. In comparison, Scab Creek and Fall Creek received 2 years of treatment, the impacts of which are less clear. Points represent the proportion of seropositive animals tested in a given year, with 95% confidence intervals (bars). Vertical dotted lines represent years in which test-and-slaughter occurred at each site. Smoother lines were fit using generalized additive models with the mgcv package [25] in R [26]. (Online version in colour.)

Experimental removal of seropositive elk took place on the Muddy Creek, Fall Creek and Scab Creek feedgrounds between 2006 and 2010. Seroprevalence decreased among yearling or older female elk from 37% to 5% over the 5 years of treatment at Muddy Creek, with approximately 50% of yearling and older females being tested. Elk were removed at Scab Creek and Fall Creek for only two years. While prevalence dropped at all three feedgrounds during treatment, prevalence among elk at Scab Creek was higher post-treatment compared to pre-treatment, and there was minimal change at Fall Creek (figure 4). Some of this variation can probably be explained by pre-treatment seroprevalence and feeding season lengths. Both Scab Creek and Fall Creek had lower pre-treatment disease prevalence than Muddy Creek, and Fall Creek feedground has the shortest duration feeding season among feedgrounds; see §3d. This experiment suggests that multiple years of test-and-slaughter are required to reduce seroprevalence but the varying treatment periods, limited pre-treatment data and different outcomes across sites all limit the strength of that conclusion. Culling based on serology results might be useful where a feedground closure is anticipated, but absent significant changes to aggregation patterns during the transmission season, depleting the pool of recovered (and still seropositive) animals could lead to more infectious contacts in subsequent years and a return to pre-treatment prevalence [58,59].

### (c) Density manipulation

A relatively low-cost method to reduce elk densities during feeding is by distributing feed over a larger area. The probability of a susceptible elk becoming infected with brucellosis is correlated with contact rate and duration of contact with infected-aborted fetuses, which both increase with local elk density and are thus elevated on feedgrounds [18]. Therefore, management strategies that reduce adult–fetus contacts [4] and adult–adult contacts [60] should lead to reduced disease transmission. In one experiment, *Brucella*-free elk fetuses were randomly placed along feedlines under high- and low-density treatments [4]. Under low-density conditions, the number of fetal contacts fell dramatically, and elk density itself dropped by 80%.

Low-density feeding has been adopted on 17 state feedgrounds, although uniform implementation at these sites has been logistically constrained. Available space for feeding differs among locations, leading to non-random selection of treatment sites, and the experience level of elk feeders, who must work a team of horses through deep snow, is variable. Both of these concerns have the potential to obscure treatment effects.

### (d) Shortened feeding season

**Figure 5.**
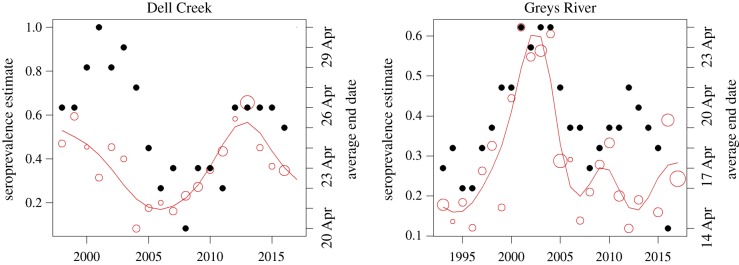
Seroprevalence estimates (empty circles, scaled to sample size) and rolling average feedground end date in the previous 8 years (solid circles) at Dell Creek and Greys River feedgrounds. Smoothed seroprevalence estimates (lines) were fit using generalized additive models in the mgcv package [25] in R [26]. (Online version in colour.)

In a comprehensive study, over 55% of the spatial variation in brucellosis seroprevalence among feedground elk was explained by the length and ending date of the supplemental feeding season using the average season end date for a feedground over the previous 8 years [61]. This also appears to be the case over time at the two sites for which there is strong longitudinal sampling (figure 5). This relationship is probably a function of abortion events driving the spread of the disease, and the timing of abortion events, which peak between March and May [23]. As with low-density feeding, WGFD has adopted earlier end dates at several feedgrounds. To mitigate the potential risk of displacing elk onto private property, targets for ‘early’ ending are based on relative snow conditions and when elk would normally depart voluntarily, as opposed to calendar date. This approach reduces risk to cattle, but limits our ability to evaluate treatment effect.

### (e) Temporary sterilization

One recently proposed intervention is the use of a gonadotropin-releasing hormone (GnRH) vaccine to temporarily sterilize female elk and bison [58,62]. In both captive and free-ranging elk, it reduces pregnancy for 1–3 years following a single dose [62,63]. It neither disrupts pregnancy upon initial delivery [63] nor affects the reproductive development of offspring [64]. In theory, it could be selectively administered to infected animals to reduce the risk of abortions in years 2 and 3; however, most abortions are thought to occur in year 1. This is further complicated by imperfect detection of brucellosis and the potential for reductions in population growth (see [58] for an in-depth discussion in the context of bison). Importantly, if transmission is driven by animals aborting in the season in which they become infected, then sterilization will not be effective. If, however, successive abortions in the years following initial infection are more important than limited captive studies have suggested, then targeted GnRH vaccination should circumvent transmission events and increase herd immunity. Many of the feedground herds are considered to be ‘over management objective’ so that some loss of reproduction could help accomplish desirable disease and population goals, but careful consideration of which animals to target for vaccination is required.

## 4. Conclusion and future directions

Prevalence of brucellosis remains high in fed elk and has become self-sustaining in unfed herds too. Past control efforts at feedgrounds, including vaccination and test-and-slaughter, have not changed the dynamics of this host–pathogen system. Current efforts, including density and feeding-duration manipulations, have yet to be thoroughly assessed but from current serology they seem unlikely to resolve brucellosis by themselves. Temporary sterilization is an option, but it remains unclear how this could or should be implemented. This leads to the inevitable discussion of closing feedgrounds and/or reducing feeding operations. Neighbouring states have closed feedgrounds in the past, although none have operated on the same scale as Wyoming and so serious questions remain. Could the winter range support the current elk population without supplemental feeding? If not, should feedground closure be combined with culling and how would that be received by the public? If feedgrounds were closed, where would elk spend the winter? How much additional hazing would be necessary to achieve the same spatio-temporal separation between elk and cattle? If some feedgrounds were phased out before others, would that cause larger elk aggregations at the remaining ones? Are private refugia and irrigated fields essentially functioning as feedgrounds outside of management control? Answers to such questions have real implications for not only disease management but also ranching, hunting and guiding, non-consumptive tourism, highway safety and animal welfare ethics.

So far, continued feeding has mollified most stakeholders within Wyoming. However, changing disease patterns, including the arrival of chronic wasting disease (CWD) (see [65]), might shift the balance of opinion. CWD has now been detected in mule deer (*Odocoileus hemionus*) or moose (*Alces alces*) in two of the seven elk herd units containing feedgrounds, although no cases have yet been reported in elk in those areas. Unlike brucellosis, CWD is fatal in cervids. It is transmitted both directly and indirectly [66], persists in the environment [67] and the known transmission routes continue to expand [68–72]. Feedgrounds are poised to concentrate infectious material and spread the disease among elk and other susceptible ungulates that use or pass through those areas. CWD probably represents a much bigger threat to cervid populations in the GYE than brucellosis, but has not yet been shown to infect cattle [73] or humans [74], although the possibility cannot be ruled out. Unless an efficacious vaccine against CWD becomes available, the only useful applications of feedgrounds to CWD management could be surveillance and removal of infected animals, the benefits of which would probably be outweighed by the risks of concentrating and spreading CWD.

Despite the risks, the continued adaptive management of WY feedgrounds, together with experimental closures, will enhance knowledge on disease dynamics and the consequences of terminating feeding should that be deemed necessary. Winter feeding in the southern GYE draws in most of the local elk population to fixed locations for at least three months each year. By using baited corral traps and restraint chutes at these feedgrounds, it is possible to handle, mark and sample hundreds of elk each winter. A before–after control-impact study could assess the interactive effects of supplemental feeding on host stress, the microbiome, reproduction, immune function and disease susceptibility, with marked elk sampled for several years before and after experimental closures. Faecal pellets and blood from restrained elk can be used to determine relative stress levels, pregnancy, bacterial killing ability, cytokine levels, genetics and brucellosis serostatus. Fine-resolution spatial data on wolf and elk movement could elucidate predator–host–disease interactions during winter that catalyse or antagonize brucellosis transmission. Together with the results of previous and ongoing telemetry studies, such information would provide early insight into the effects of feedground closures on elk population, movement and disease ecology.

## Supplementary Material

R code for seroprevalence of unfed herds (fig 3)

## Supplementary Material

R code for seroprevalence at test and slaughter feedgrounds (fig 4)

## Supplementary Material

R code for feeding end dates in relation to seroprevalence at feedgrounds (fig 5)

## Supplementary Material

R code. Function to create prevalence estimates by location and year.

## Supplementary Material

R code. Function to create a smoother line using generalised additive models.

## Supplementary Material

Unfed elk herd serology data.

## Supplementary Material

Test and slaughter serology data.

## Supplementary Material

Feeding season end date data.

## Data accessibility

The datasets and code supporting this article have been uploaded as part of the electronic supplementary material.
